# A new species of *Hypodematium* (Hypodematiaceae) from China

**DOI:** 10.3897/phytokeys.92.21815

**Published:** 2018-01-15

**Authors:** Xiaojuan Li, Jianxiu Li, Fanyun Meng

**Affiliations:** 1 Beijing Key Laboratory of Protection and Application of Chinese Medicinal Resources, Beijing Normal University, Beijing 100875, China; 2 Faculty of Geographical Science BNU, Beijing Normal University, Beijing 100875, China; 3 Shandong Hongjitang Museum, Jinan 250100, China; 4 Shandong University of Traditional Chinese Medicine, Jinan 250014, China

**Keywords:** *Hypodematium confertivillosum*, *Hypodematium crenatum*, *Hypodematium glanduloso-pilosum*, spore ornamentation, SEM

## Abstract

*Hypodematium confertivillosum* J.X.Li, F.Q.Zhou & X.J.Li, **sp. nov.**, a new species of *Hypodematium* from Shandong, China, is described and illustrated. It is similar to *H. crenatum* (Forssk.) Kuhn & Decken and *H. glanduloso-pilosum* (Tagawa) Ohwi, but differs greatly from them by its abaxial fronds sparsely covered with rod-shaped glandular hairs, its adaxial fronds without rod-shaped glandular hairs and spore reniform, with verrucate processes, surface with distinct finely lamellar rugae ornamentation. The description, photographs and a key to *H. confertivillosum* as well as their notes are provided.

## Introduction

Described in 1833, *Hypodematium* Kunze is the only genus of Hypodematiaceae Ching (Ching 1975). Iwatsuki (1964) reviewed the genus and recognised four species including one subspecies. Recently, more than 16 species of *Hypodematium*, mainly distributed in subtropical and temperate areas of Asia and Africa, have been established (Shing et al. 1999). China, with 12 species of *Hypodematium*, is regarded as the centre of distribution for this genus (Zhang and Iwatsuki 2013). The genus is characterised by a distinctive swollen scaly stipe base and grows only on limestone habitat (Zhang and Iwatsuki 2013). Previous research on systematics and palynology of *Hypodematium* (Ching 1935, 1940, 1963, 1975, 1978a, b, Li et al. 1988, Shing et al. 1999, Zhou et al. 1999, Wang et al. 2010, Zhang and Iwatsuki 2013) provided an important background that allowed the recognition of the species new to science.

## Materials and methods

The voucher specimens of the new species were collected from Tashan mountain, China and deposited in PE (herbaria acronyms according to Thiers 2016).

Scanning electron microscopy (SEM) was used to document the micromorphology of spore and fronds. Samples were dehydrated and were then placed on aluminium stubs using double-sided adhesive tape and sputter coated with gold in a Hitachi E-1010 Ion Sputter Coater, following Wen and Nowicke (1999). The materials were subsequently observed and photographed under a SUPRATM55 scanning electron microscope.

## Taxonomy

### Hypodematium confertivillosum

Taxon classificationPlantaePolypodialesHypodematiaceae

J.X.Li, F.Q.Zhou & X.J.Li sp. nov.

urn:lsid:ipni.org:names:77174973-1

#### Diagnosis.

*Hypodematium confertivillosum* J. X. Li, F. Q. Zhou & X. J. Li is similar to *H. crenatum* (Forssk.) Kuhn & Decken and *H. glanduloso-pilosum* (Tagawa) Ohwi, from which it differs greatly by its abaxial fronds sparsely covered with rod-shaped glandular hairs, its adaxial fronds without rod-shaped glandular hairs and spore reniform, with verrucate processes, surface with distinct finely lamellar rugae ornamentation.

#### Type.

**Figure 1.**
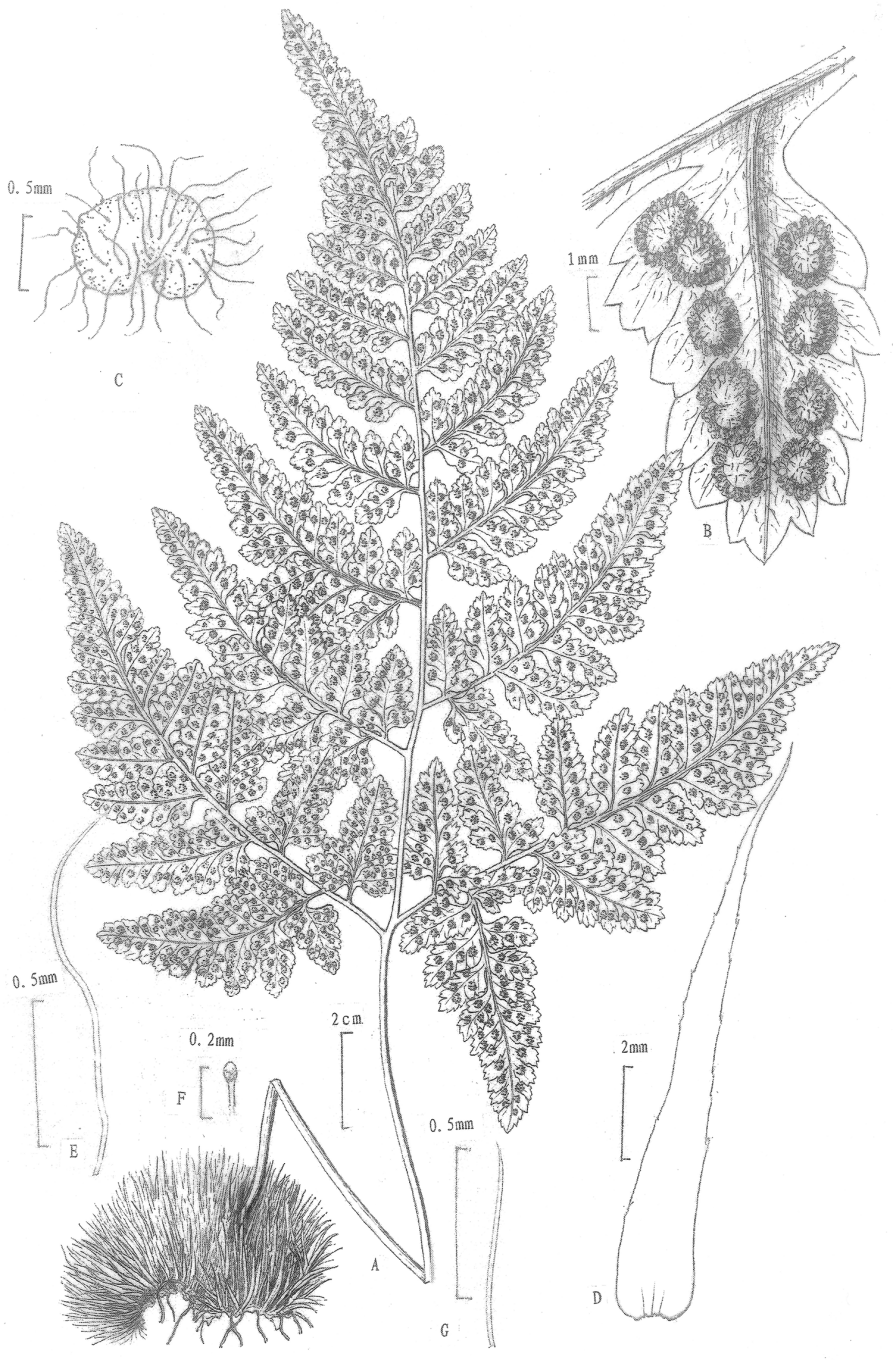
*Hypodematium confertivillosum* J.X.Li, F.Q.Zhou & X.J.Li, sp. nov. **A** Habit **B** Sori on the abaxial surface of pinnules **C** Indusium with long hairs **D** Rhizome and stipe base scales **E** Long hairs from the abaxial surface of fronds **F** Rod-shaped glandular hairs from the abaxial surface of fronds **G** Hairs from the adaxial surface of fronds (Drawn by Y. B. Sun & J. X. Li).

China. Shandong Province: Linyi City, Fei County, Tashan Mountain, limestone rocks, 35°33'59.76"N, 117°51'29.51"E, 500–700 m a.s.l., 15 September 1982, J. X. Li 02025 (Holotype: PE, Isotype: SDCM). Figure 1.

#### Description.

Plants 21–32 cm tall. Rhizomes creeping; densely scaly together with stipe base, scales reddish-brown, lustrous, linear-lanceolate, 10–12 × 1–2 mm, membranaceous, margin subentire, apex acuminate. Fronds approximate; stipe stramineous, 7–17 cm × 1–1.2 mm, nearly glabrous upward; laminae pentagonal, 12–17 × 12–14 cm, 3-pinnate-pinnatifid, base round-cordate, apex acuminate and pinnatifid; pinnae 10–12 pairs, slightly oblique, lower 2 pairs sub-opposite, 3–4 cm apart, upper pairs alternate; basal pinnae largest, deltoid-oblong, 10–11 × 8–8.5 cm, 2-pinnate-pinnatifid, base cordate, pinnae tapered; pinnules 6–8 pairs, anadromous, alternate, slightly oblique, acroscopic ones smaller, proximal basiscopic pair largest, ovate-triangular, 5 × 2–3 cm, shortly stalked, base cuneate, pinnae tapered, pinnate-pinnatifid; ultimate pinnules oblong, 8–10 × 4–6 mm, apex obtuse, pinnatifid; lobe oblong, apex obtuse, margins obtuse-serrate; second and upper pairs of pinnae gradually shorter, lanceolate or oblong-lanceolate, 2-pinnate-pinnatifid, base rounded-cuneate or shallowly cordate, with a short stalk, apex shortly acute. Veins obvious on both surfaces, pinnate, simple, ending at margin. Laminas chartaceous, fronds densely covered with long grey hairs adaxially, fronds abaxial surface, rachis and costae densely covered with long grey hairs and sparsely mixed with rod-shaped glandular hairs. Sori round, dorsal, 1–4 per segment; indusia reniform, pale grey, membranaceous, densely covered with grey hairs. Spores reniform, with verrucate processes, surface with distinct finely lamellar rugae ornamentation.

#### Distribution.

This species is known only from the area around the type locality in Tashan, Shandong.

#### Ecology.

Usually growing in limestone crevices of xeric areas.

#### Discussion.

**Figure 2.**
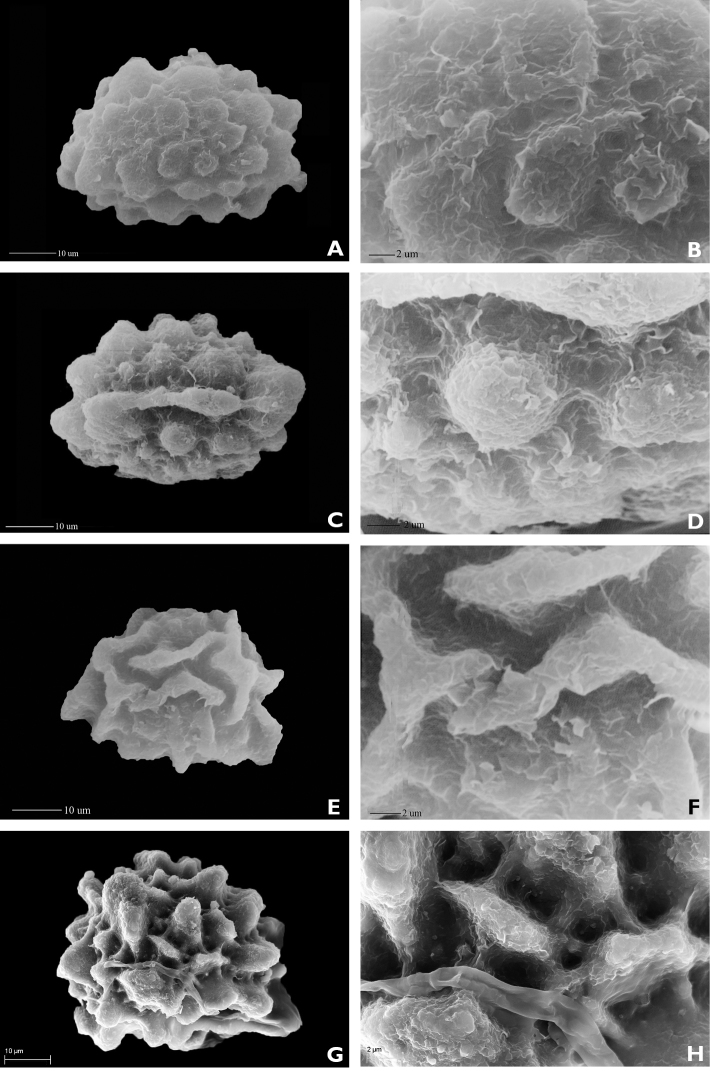
Spore morphologies of three *Hypodematium* species (SEM). **A** Spore in equatorial view of *H. confertivillosum* (1500×) **B** Detail of spore in equatorial view of *H. confertivillosum* (5000×) **C** Spore in polar view of *H. confertivillosum* (1500×) **D** Detail of spore in polar view of *H. confertivillosum* (5000×) **E** Spore in equatorial view of *H. crenatum* (1500×) **F** Detail of spore in equatorial view of *H. crenatum* (5000×) **G** Spore in equatorial view of *H. glanduloso-pilosum* (1500×) **H** Detail of spore in equatorial view of *H. glanduloso-pilosum* (5000×).

The perispore is an important trait for identifying species under the scanning electron microscopy (Liu and Li 1999) and it contributes to the discovery of some new species, for example *Dryopteris guanchica* (Jermy 1980). There are significant differences between the perispore of *H. confertivillosum* that has verrucate processes, surface with distinct finely lamellar rugae ornamentation, *H. crenatum* having curved long ridges, surface with fine striae ornamentation and *H. glanduloso-pilosum* having tuberculate-massive ornamentation, providing an important micromorphological basis for establishment of the new species *H. confertivillosum*. A comparison of *H. confertivillosum*, *H. crenatum*, and *H. glanduloso-pilosum* is given in Table 1 and Figure 2.

**Table 1.** Comparison of spore morphological features amongst three species of *Hypodematium*.

Species name	Size (μm)	Ornamentation of perispore SEM	Locality and voucher	Figure 2
*H. confertivillosum*	40.8×52.6	Verrucate processes, surface with finely lamellar rugae	Shandong J.X. Li 02025 PE	A–D
*H. crenatum*	46.1×50.3	Curved long ridges, surface with fine striae	Guangxi R.H. Zhou 0013-1 PE	E–F
*H. glanduloso-pilosum*	48.2×53.6	Tuberculate-massive	Shandong J.X. Li 96-035 SDCM	G–H

**Figure 3.**
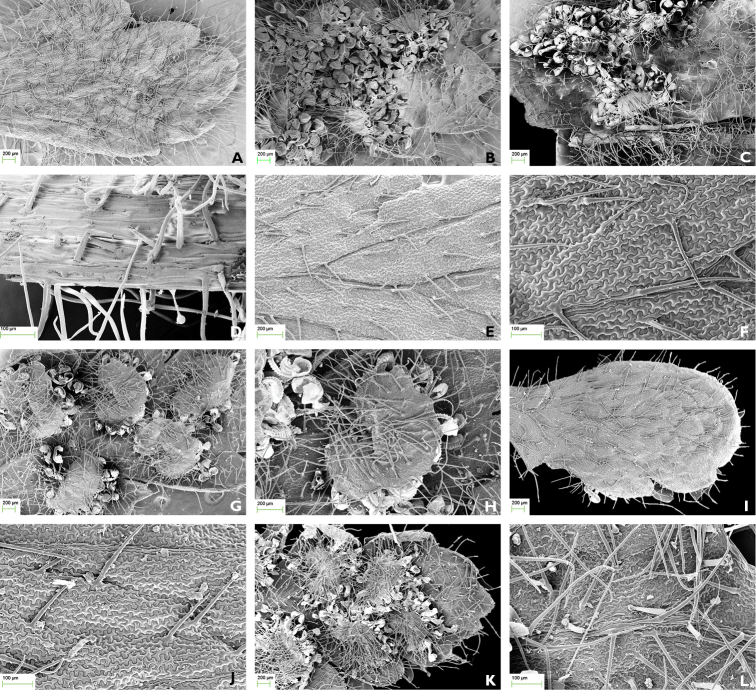
The fronds and rachis of *H. confertivillosum*, *H. crenatum* and *H. glanduloso-pilosum* (SEM). **A** *H. confertivillosum* fronds densely covered with long hairs adaxially (30×) **B** *H. confertivillosum* fronds and indusia densely covered with long hairs and sparsely rod-shaped glandular hairs abaxially (30×) **C** *H. confertivillosum* fronds and costae densely covered with long hairs and sparsely rod-shaped glandular hairs abaxially (30×) **D** *H. confertivillosum* costae densely covered with long hairs and sparsely rod-shaped glandular hairs abaxially (160×) **E** *H. crenatum* fronds sparsely covered with acicular hairs adaxially (60×) **F** Close-up view of *H. crenatum* fronds covered with acicular hairs adaxially (140×) **G** *H. crenatum* fronds and indusia densely covered with long hairs abaxially (30×) **H** Close-up view of *H. crenatum* indusia covered with long hairs abaxially (60×) **I** *H. glanduloso-pilosum* fronds densely covered with acicular hairs and rod-shaped glandular hairs adaxially (30×) **J** Close-up view of *H. glanduloso-pilosum* fronds covered with acicular hairs and rod-shaped glandular hairs adaxially (140×) **K** *H. glanduloso-pilosum* fronds and indusia densely covered with long hairs and rod-shaped glandular hairs abaxially (30×) **L** Close-up view of *H. glanduloso-pilosum* fronds covered with long hairs and rod-shaped glandular hairs abaxially (140×)

It is commonly believed that *Hypodematium*, a very special group, has different types of glandular hairs and non-glandular hairs, which is an important basis for the identification and classification of species of *Hypodematium* (Zhang and Iwatsuki 2013). *Hypodematium confertivillosum* fronds are sparsely covered with rod-shaped glandular hairs abaxially, but its adaxial fronds without rod-shaped glandular hairs; *H. crenatum* fronds are sparsely covered with acicular hairs adaxially, densely covered with long hairs abaxially and without rod-shaped glandular hairs on both surfaces. *Hypodematium glanduloso-pilosum* fronds are mixed, densely covered with acicular and rod-shaped glandular hairs adaxially and long hairs and rod-shaped glandular hairs abaxially. Therefore, the types of hair and the degree of density of different types of hair support the establishment of the new species of *H. confertivillosum*. A comparison of *H. confertivillosum*, *H. crenatum*, and *H. glanduloso-pilosum* is given in Table 2 and the taxonomic key below (adapted from Zhang and Iwatsuki 2013), and Figure 3.

**Table 2.** Comparison of fronds and indusia in three species of *Hypodematium*.

Species name	Adaxial fronds		Abaxial fronds		Rachis and costae		Indusia		Holotype, voucher and gatherer	Figure 3
	**Non-glandular hairs**	**Glandular hairs**	**Non-glandular hairs**	**Glandular hairs**	**Non-glandular hairs**	**Glandular hairs**	**Non-glandular hairs**	**Glandular hairs**		
*H. confertivillosum*	Densely covered with long grey hairs	Absent	Densely covered with long grey hairs	Sparsely rod-shaped glandular hairs	Densely covered with long grey hairs	Sparsely rod-shaped glandular hairs	Densely covered with long grey hairs	Sparsely rod-shaped glandular hairs	Holotype J. X. Li 02025	A–D
*H. crenatum*	Sparsely acicular hairs	Absent	Densely covered with long grey hairs	Absent	Densely covered with long grey hairs	Absent	Densely covered with long grey hairs	Absent	Voucher R. H. Zhou 0013-1	E–H
*H. glanduloso-pilosum*	Densely covered with acicular hairs	More rod-shaped glandular hairs	Densely covered with long grey hairs	Densely covered with rod-shaped glandular hairs	Densely covered with long hairs	Densely covered with rod-shaped glandular hairs	Densely covered with grey hairs	Densely covered with rod-shaped glandular hairs	Voucher J. X. Li 96-035	I–L

### Taxonomic key to the species of *Hypodematium*

**Table:** 

1	Fronds not covered with rod-shaped glandular hairs adaxially	**2**
–	Fronds covered with rod-shaped glandular hairs and long grey hairs on both surfaces; perispore with tuberculate-massive ornamentation	***H. glanduloso-pilosum***
2	Fronds sparsely covered with rod-shaped glandular hairs abaxially; perispore with verrucate processes, surface with finely lamellar rugae ornamentation	***H. confertivillosum***
–	Fronds not covered with rod-shaped glandular hairs abaxially; perispore with curved long ridges, surface with fine striae ornamentation	***H. crenatum***


## Supplementary Material

XML Treatment for Hypodematium confertivillosum
